# Genetic Method for Labeling Electrically Coupled Cells: Application to Retina

**DOI:** 10.3389/fnmol.2015.00081

**Published:** 2016-01-07

**Authors:** Mu Qiao, Joshua R. Sanes

**Affiliations:** Center for Brain Science and Department of Molecular and Cellular Biology, Harvard University, CambridgeMA, USA

**Keywords:** electrical synapse, gap junction, retina, horizontal cells, peptide transporter, PEPT2

## Abstract

Understanding how the nervous system functions requires mapping synaptic connections between neurons. Several methods are available for imaging neurons connected by chemical synapses, but few enable marking neurons connected by electrical synapses. Here, we demonstrate that a peptide transporter, Pept2, can be used for this purpose. Pept2 transports a gap junction-permeable fluorophore-coupled dipeptide, beta-alanine-lysine-*N*-7-amino-4-methyl coumarin-3-acid (βALA). Cre-dependent expression of *pept2* in specific neurons followed by incubation in βALA labeled electrically coupled synaptic partners. Using this method, we analyze light-dependent modulation of electrical connectivity among retinal horizontal cells.

## Introduction

Because neurons communicate with each other primarily through synapses, mapping patterns of synaptic connectivity is an essential step in understanding how neural circuits function. Synapses are of two types: chemical, in which neurotransmitter released by the presynaptic cell signals to its postsynaptic partner, and electrical, in which currents pass directly from cell to cell through gap junctions (Siegelbaum and Kandel, 2013). To date, chemical synapses have received the vast majority of attention (Sanes and Yamagata, 2009; Yogev and Shen, 2014). In contrast, patterns of electrical connectivity have been studied in relatively few cases (White et al., 1986; Volgyi et al., 2009; Varshney et al., 2011). In part, this inattention stemmed from the belief that electrical synapses were relatively rare in vertebrates. Over the past decade, however, it has become apparent that they are in fact numerous and play diverse roles in developing and adult animals (Connors and Long, 2004; Hormuzdi et al., 2004; Bloomfield and Volgyi, 2009; Cook and Becker, 2009; Li et al., 2012; Yu et al., 2012; Apostolides and Trussell, 2013; Zhu et al., 2013). It is therefore essential to redress the balance.

One obstacle to the study of electrical synaptic connections -perhaps both a cause and an effect of the scant attention they have been paid- is that few methods are available for mapping them in the intact nervous system. Electrical connectivity is most often assessed by impaling a neuron with a microelectrode for injection of a dye that diffuses through gap junctions (Vaney, 1991; Mills and Massey, 1994; Hoshi et al., 2006). This approach is laborious, and difficult to apply to cells that are small, fragile, or deeply buried in tissue. An alternative, intracellular recording of potentials evoked by stimulating a neighboring cell is even more difficult, because it faces the added obstacle of requiring paired recordings. Light microscopic immunohistochemical localization of individual electrical synapses is infeasible, both because gap junctions are often near the limit of optical resolution and because no single component is known that marks all and only electrical synapses. Electron microscopy provides sufficient resolution, but is hampered by the need to connect synapses to cells of origin through reconstruction from serial sections, which is laborious and currently applicable only to small volumes.

**FIGURE 1:**
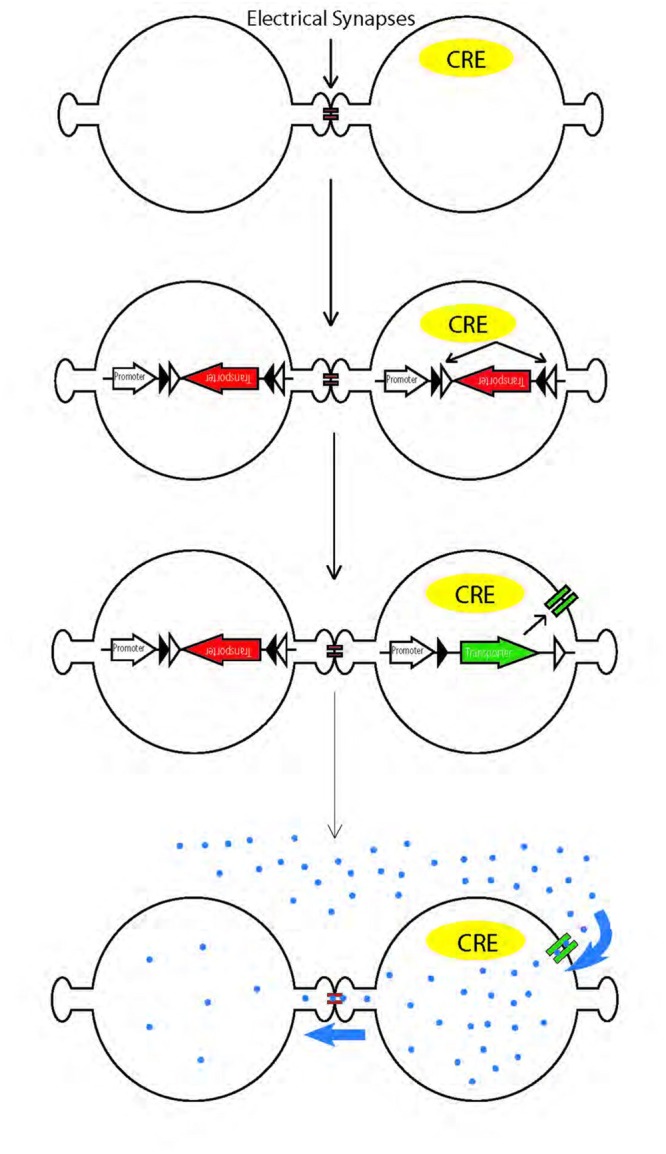
**Schematic illustration of genetic method for labeling electrically coupled cells from Cre-positive cells.** A channel or transporter is expressed in Cre-positive cells, allowing them to take up gap junction permeable fluorescent substrates. The substrates diffuse through gap junctions to label coupled cells.

Recently, mapping of chemical synaptic connectivity has been aided by a variety of genetic methods for labeling synaptically connected neuronal pairs (Wickersham et al., 2007; Feinberg et al., 2008; Beier et al., 2011; Lo and Anderson, 2011). Parallel methods for mapping electrical synaptic connectivity could provide similar benefits. Recently, one such method was introduced, in which an esterase is targeted to specific cells; it acts on a membrane-permeable substrate to generate a fluorophore that can pass through gap junctions (Tian et al., 2012). Here, we report an alternative method, in which we use cre recombinase-dependent vectors to target a peptide transporter to the membrane of cre-expressing cells *in vivo*. We then incubated the tissue with a membrane-impermeable fluorophore-conjugated dipeptide that can, once inside the cell, pass through gap junctions from the transporter-expressing cell to electrically connected neighbors (**Figure 1**).

## Results

### Pept2 Mediates Labeling of Electrically Coupled Cells

**FIGURE 2:**
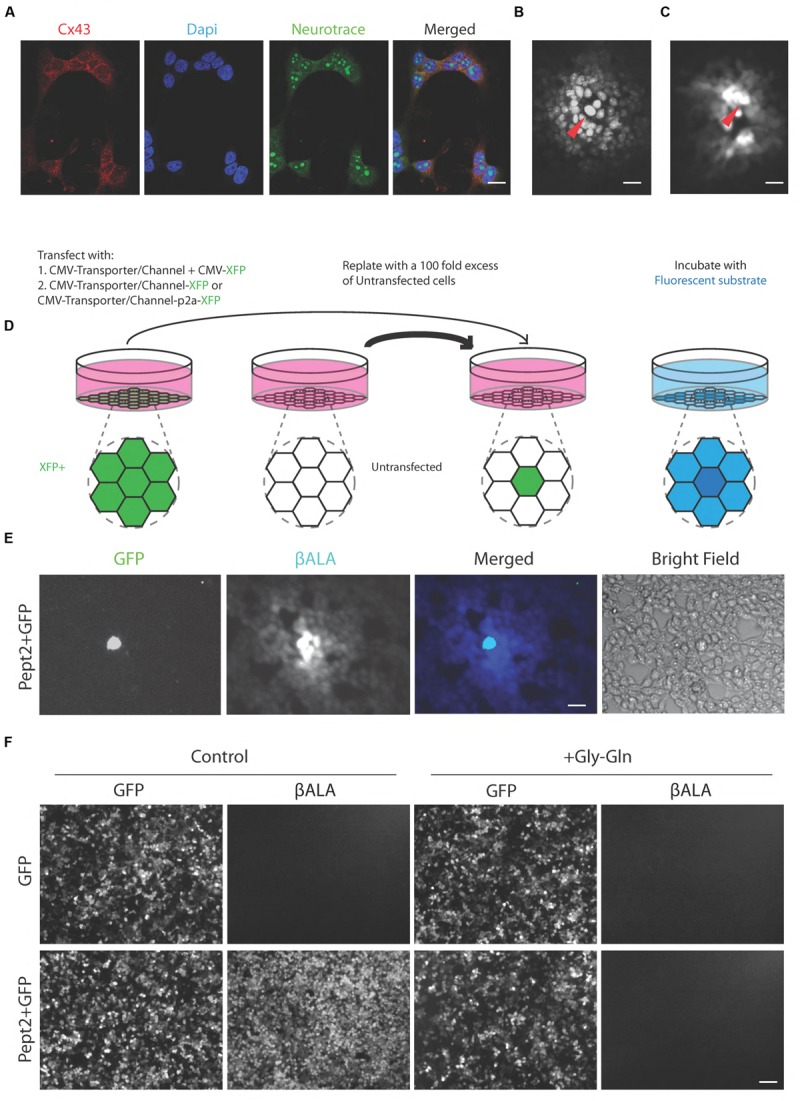
**Pept2 method enables labeling gap junction-coupled cells. (A)** Cultured HEK cells stained with antibody against connexin 43 (Cx 43). Dapi shows cell nucleus and Neurotrace stains cytoplasm. Connexin 43 is enriched at the cell membrane between neighboring cells. **(B)** HEK cells injected with neurobiotin and stained with Texas red-streptavidin. Red arrow indicates the injected cell. **(C)** HEK cells injected with βALA. Red arrow indicates the injected cell. **(D)** Schematic illustration of the method for testing channels or transporters capable of importing small fluorescent substrate that can permeate gap junctions. **(E)** HEK cells were transfected with GFP and Pept2, then mixed with a 100-fold excess of untransfected cells and incubated with βALA. βALA diffused from GFP positive cells to adjacent coupled cells. **(F)** HEK cells were transfected with GFP, GFP + Pept2 or Pept2-GFP. Uptake of βALA was eliminated by Pept2 inhibitor Gly–Gln. Scale bars in **(A–C,E)**: 20 μm; in **(F)**: 100 μm.

We used a human embryonic kidney cell line, HEK293, to test transporters and channels that would facilitate entry of membrane-impermeable but gap junction-permeable fluorescent molecules into cells. HEK cells have been reported to express connexin 43 and to form numerous gap junctions when grown as a confluent monolayer (Gemel et al., 2004; Johnson et al., 2013; Patel et al., 2014); we confirmed expression of connexin 43 immunohistochemically (**Figure 2A**) and we confirmed strong coupling by microinjection of neurobiotin, which is widely used to monitor coupling in tissue (**Figure 2B**).

We tested five protein/substrate pairs. Three were channels, TRPV1, TRPA1, and P2X7. All three are non-selective cation channels that are permeable to fluorescent molecules such as, Yo-pro-1 (TRPV1 and TRPA1) and ethidium (P2X7), (Meyers et al., 2003; Chen et al., 2009; Browne et al., 2013). We also tested the fluorescent dye Po-pro-1, which is closely related to Yo-pro-1. The other two proteins were related drug and peptide transporters, Pept1 and Pept2, both of which translocate the AMCA-labeled dipeptides beta-Ala-Lys (βALA) and D-Ala-Lys (Dieck et al., 1999; Groneberg et al., 2001). Ethidium and Po-pro-1 are known to cross gap junctions (Hoshi et al., 2006; Kanaporis et al., 2011) and we used microinjection to show that the same is true for βALA (**Figure 2C**).

In each case, we introduced the channel or transporter plus a fluorescent protein (XFP) by transfection into HEK293 cells, grew them for 1–2 days, then replated them with a 100-fold excess of unlabeled cells. The XFP was chosen to be distinguishable from the fluorophore to be tested. Once the cells reached confluency, we incubated them with the fluorophore. We activated TRPV1, TRPA1, and P2X7 by co-incubation with their ligands, capsaicin, allyl isothiocyanate (AITC) and ATP, respectively. We assessed uptake and transfer by observing substrate in XFP-labeled cells and neighboring XFP-negative cells, respectively (**Figure 2D**).

Signals in cells expressing TRPV1, TRPA1, and P2X7 channels were very dim and fluorophore was rarely detected in neighboring untransfected cells. In contrast, both Pept1 and Pept2 supported robust uptake of βALA, which was then transferred to neighboring cells. Of the two, Pept2 was superior, perhaps reflecting its higher affinity (Daniel and Rubio-Aliaga, 2003; Zhang et al., 2004b; Biegel et al., 2006), (**Figure 2E**). Uptake was specific in that it was negligible in untransfected cells and was completely blocked by incubation with a 200-fold excess of the competitive inhibitor, Glycine–Glutamine (Gly–Gln), (**Figure 2F**). We therefore used Pept2 for all subsequent studies reported here.

**FIGURE 3:**
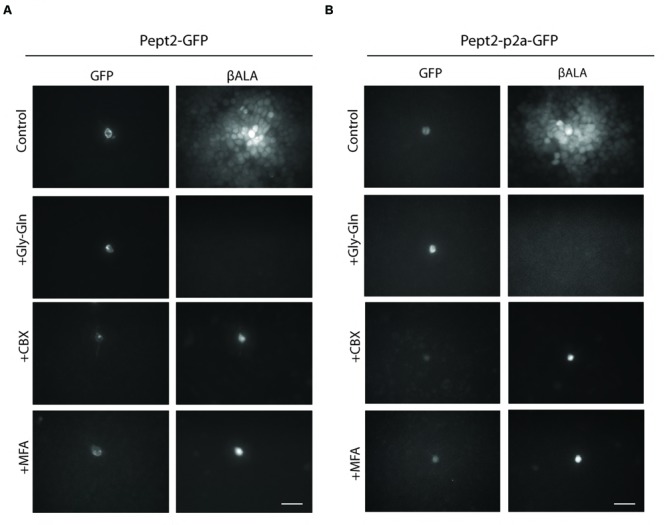
**Both Pept2-GFP and Pept2-p2a-GFP mediate βALA uptake.** HEK cells were transfected with Pept2-GFP **(A)** or Pept2-p2a-GFP **(B)**. Uptake of βALA was blocked by Pept2 inhibitor Gly–Gln. Diffusion of βALA to coupled cells was blocked by gap junction blockers MFA and CBX. Scale bar: 20 μm.

We generated two vectors to express both Pept2 and GFP from a single transcript. In one, *Pept2-GFP*, we fused GFP directly to the C-terminal of Pept2. In the other, *Pept2-p2a-GFP*, the two proteins were separated by the self-cleaving p2a sequence. When tested in HEK 293 cells as above, both constructs worked as efficiently as Pept2 alone. In both cases, uptake was inhibited by Gly–Gln and transfer from GFP-expressing to GFP-negative cells was inhibited by carbenoxolone (CBX) and meclofenamic acid (MFA), two widely used gap junction blockers (Li et al., 2012; Yu et al., 2012) (**Figures 3A,B**).

### Quantitative Measurement of Gap Junction Strength

**FIGURE 4:**
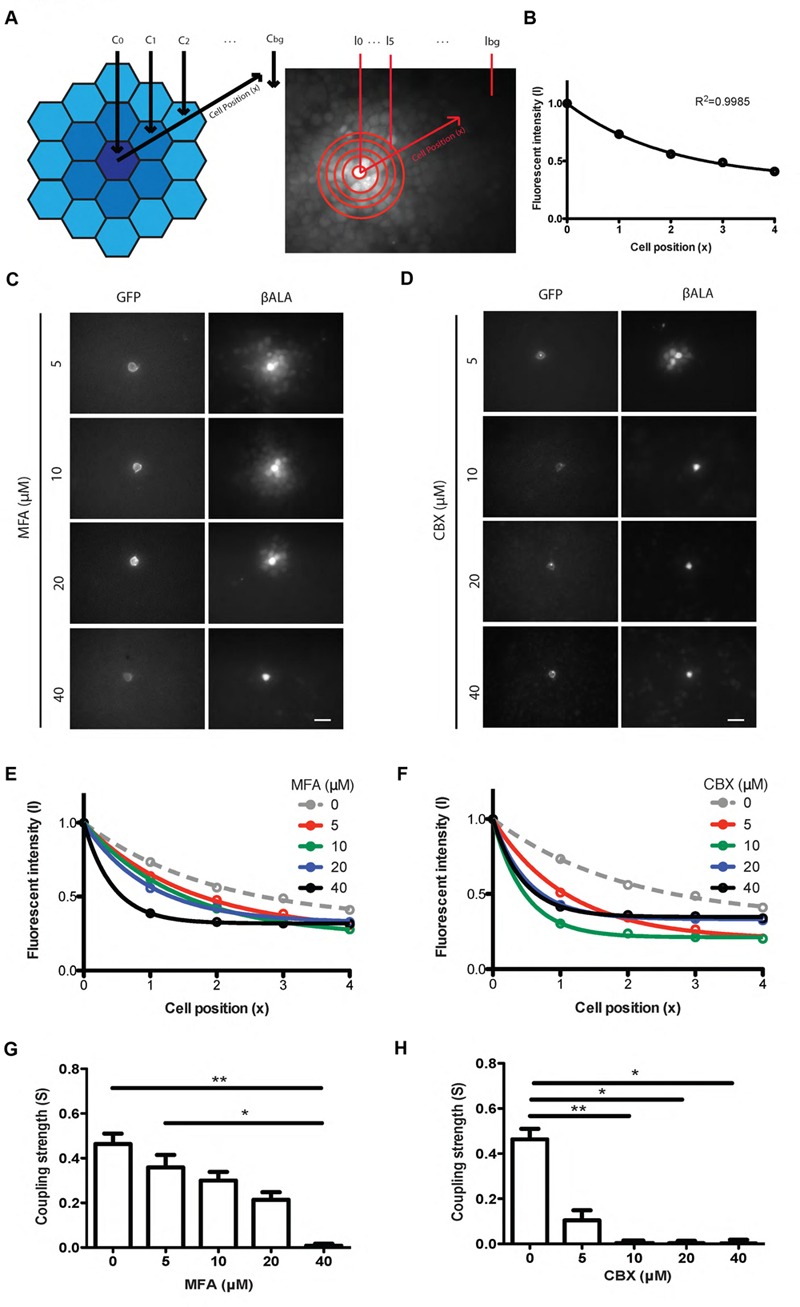
**Pept2 method enables quantification of gap junction strength between cultured cells. (A)** Schematic illustration and measurement of the βALA signals for quantification of gap junction strength. **(B)** βALA signals from the probe cell to coupled cells follow an exponential decay. **(C)** Inhibition of βALA diffusion between gap junction-coupled cells by MFA at indicated concentrations. **(D)** Inhibition of βALA diffusion between gap junction-coupled cells by CBX at indicated concentrations.**(E)** Decay curves fitted from results in **(C)**.**(F)** Decay curves fitted from results in **(D)**. **(G)** Coupling strength S calculated based on curves in **(E)**. ^∗^*p* < 0.05; ^∗∗^*p* < 0.01, *n* = 5 probe cells for each condition. Error bars are SEM. **(H)** Coupling strength S calculated based on **(F)**. ^∗^*p* < 0.05; ^∗∗^*p* < 0.01, *n* = 5 probe cells for each condition. Error bars are SEM. Scale bars in **(C,D)**: 20 μm.

To quantify the strength of gap junctional coupling, we developed an analysis based on the equation that describes the diffusion of βALA from the probe cell to the coupled cells (see Materials and Methods). We assume that the fluorescent intensity of βALA is proportional to the concentration of βALA. Therefore, at steady state, when the concentration of βALA in each cell no longer changes with time, the fluorescent signal of βALA from the probe cell to coupled cells follows an exponential decay (**Figure 4A**). We assayed diffusion patterns after 1, 2, 4, and 6 h of incubation and found no obvious difference between those of 4 and 6 h, suggesting that after 4 h of incubation, diffusion of βALA from the probe cell to the coupled cells was close to the steady state. Consistent with the result, the labeling pattern was well described by the diffusion equation following 4 h of incubation (**Figure 4B**).

To test the ability of this method to detect changes in coupling, we varied the concentration of the gap junction blockers, MFA and CBX. In both cases, the fluorescent intensity of βALA from the probe cell to coupled cells was well fitted by an exponential, with increasing concentrations of inhibitor leading to decreased diffusion and thus faster decay (**Figures 4C–F**). From each curve we estimated the decay constant and used it to derive coupling strength S, which reflects how strongly two adjacent cells are coupled by gap junctions (**Figures 4G,H**). Coupling was nearly abolished by 10 μM CBX and 40 μM MFA.

### Pept2-Mediated Labeling of Electrically Coupled Retinal Neurons

We chose mouse retina for tests *in vivo*, because it can be incubated with substrate as a thin, flat explant and because specific patterns of electrical coupling among its neurons have been mapped in detail (Bloomfield and Volgyi, 2009; Volgyi et al., 2009). Initial tests revealed, however, that βALA labeled a population of cells in wild-type retina, leading to signals that obscured some of the coupling we hoped to detect. Immunolabeling revealed that the labeled cells were predominantly Muller glia (glutamine synthetase and Sox9-positive; **Supplementary Figure S1A**), and uptake was abolished by co-incubation with Gly–Gln (**Supplementary Figure S1B**). These results suggested that Muller glia express *pept2*, which has been reported to be expressed by some glial populations in brain (Berger and Hediger, 1999; Dieck et al., 1999; Zimmermann and Stan, 2010). We therefore obtained targeted Pept2 null mutants (*pept2^-/-^*), which are viable and fertile (Shen et al., 2003). Uptake of βALA into Muller glia was abolished in retinas from *pept2^-/-^* mice (**Supplementary Figure S1C**). No neural phenotypes have been reported for *pept2^-/-^* (Shen et al., 2003) and we detected no changes in numbers, positions or arbors of any retinal cell types examined (**Supplementary Figure S2**). We therefore used *pept2^-/-^* mice for studies *in vivo*.

**FIGURE 5:**
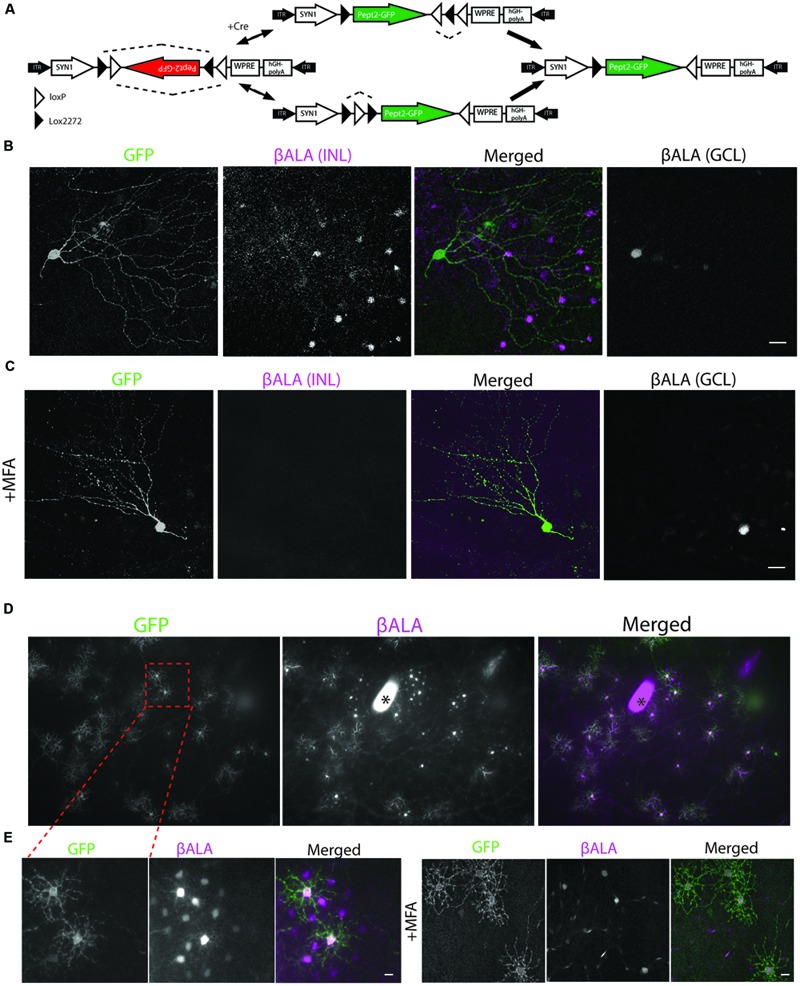
**Pept2 dependent method reveals coupled cells in the retina. (A)** Schematic of Cre-dependent AAV carrying Pept2-GFP. **(B)** βALA signals were bright in Pept2-GFP positive J-RGCs in the ganglion cell layer (GCL), and also present in coupled amacrine cells in the INL. **(C)** Diffusion of βALA to coupled amacrine cells was eliminated by MFA. Scale bars in **(B,C)**: 40 μm. **(D)** Pept2 method reveals coupling of horizontal cells. **(E)** Diffusion of βALA to coupled cells was eliminated by MFA. Scale bars in **(D,E)**: 20 μm. Asterisk indicates a blot on the cover slide.

To express Pept2 in cre-expressing neurons, we generated the adeno-associated virus (AAV) serotype 2, AAV-DiO-Pept2-GFP in which a double-floxed inverted open-reading-frame (DiO) sequence renders expression of a Pept2-GFP fusion cre-dependent (**Figure 5A**). To ensure that labeling was sparse, we used a mouse line in which one retinal ganglion cell (RGC) type, J-RGCs, express Cre fused with estrogen receptor [CreER; (Kim et al., 2008)]. In this line, recombinase activity is induced by tamoxifen in a dosage-dependent fashion. We infected mouse retina with high-titer AAV-DiO-Pept2-GFP and administered a low dose of tamoxifen 10 days later so that Pept2 would be expressed in small numbers of J-RGCs.

GFP-expressing J-RGCs were readily recognizable by their strikingly asymmetric dendritic arbors (**Figure 5B**). Coupling partners of J-RGCs were amacrine cells in the inner nuclear layer (INL) but not other RGCs, consistent with coupling patterns of presumptive J-RGCs determined by microinjection (Hoshi and Mills, 2009; Volgyi et al., 2009). MFA (100 μM) blocked transfer of βALA to the coupled amacrine cells (**Figure 5C**), indicting that βALA had been transferred from J-RGCs via electrical synapses. Thus, the Pept2 method enables labeling of electrically coupled neurons in mouse retina.

We also introduced Pept2 into horizontal cells, which are electrically coupled to each other in many species, including mice (Kaneko, 1971; Dacheux and Raviola, 1982; Mills and Massey, 1994; Bloomfield et al., 1995; Dacey, 1999; Dowling, 2012). For this purpose we injected AAV-DiO-Pept2-GFP subretinally into Sdk2-CreER; *pept2^-/-^* mice; Sdk2 is a synaptic recognition molecules that is expressed by horizontal cells as well as by specific populations of RGCs and amacrine cells (Krishnaswamy et al., 2015).

Probe (GFP-positive) cells were strongly labeled by βALA, and a set of neighboring cells were labeled less strongly. Both probe and coupled cells were identifiable as horizontal cells based on their size, shape, and mosaic arrangement (**Figure 5D**) and labeling with anti-calbindin (see below). As with J-RGCs, uptake by probe cells persisted but transfer was abolished in the presence of MFA (100 μM), confirming that transfer reflected gap junctional coupling (**Figure 5E**). Together, these results demonstrate that the Pept2 method can be used to detect electrically coupled neurons *in vivo*.

### Light-Dependent Electrical Coupling of Horizontal Cells

**FIGURE 6:**
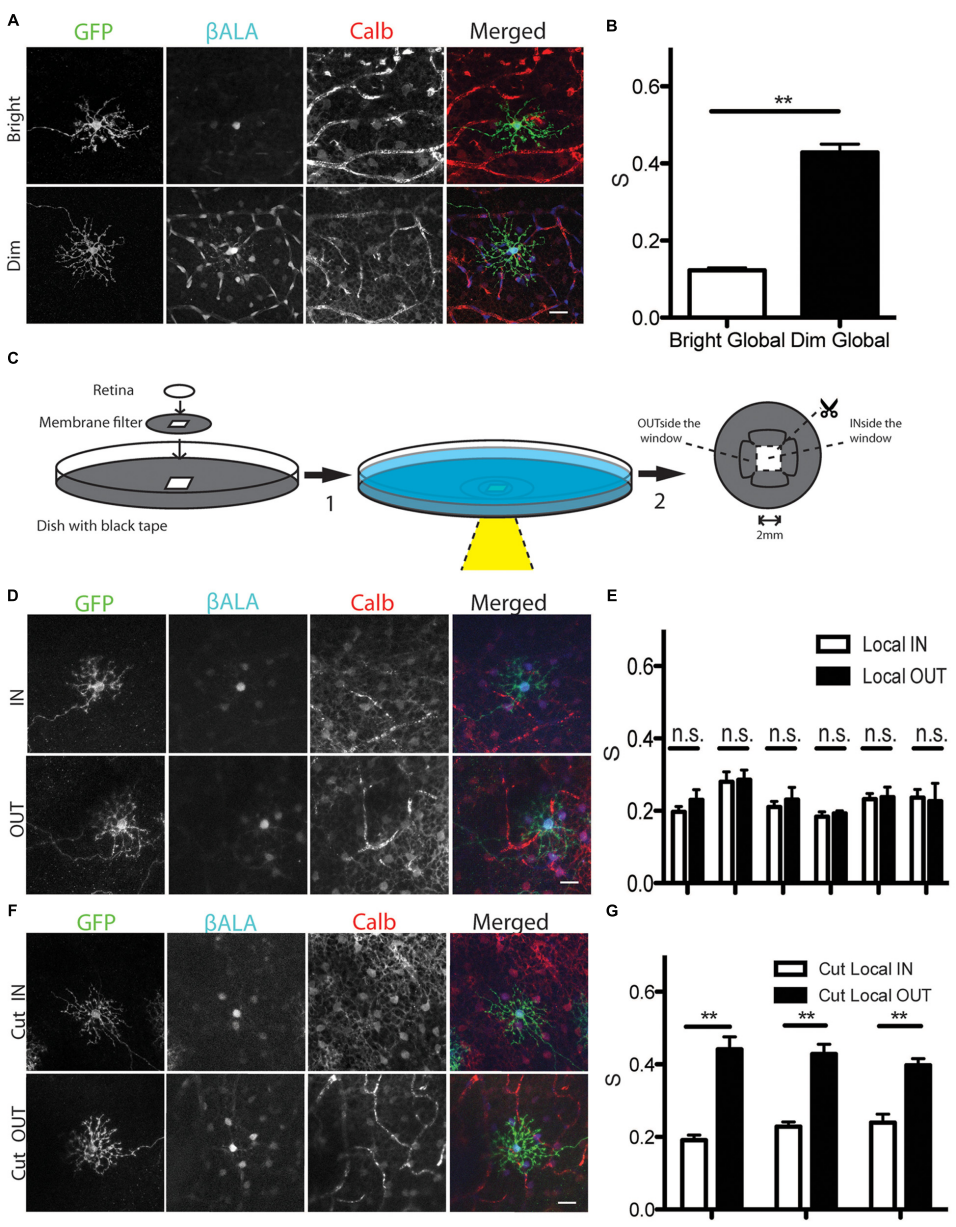
**Analysis of light dependent electrical coupling between horizontal cells. (A)** Coupling between horizontal cells is stronger under dim light than under bright light. Horizontal cells are identified by the marker calbindin (Calb). Blood vessels are also stained by the secondary antibody against mouse Calb primary antibody, but can be easily distinguished from horizontal cells. **(B)** Quantification of electrical coupling strength S in **(A)**. ^∗∗^*p* < 0.01, *n* = 73 starter cells in bright light, *n* = 45 starter cells in dim light. Error bars are SEM. **(C)** Schematic of assays to measure gap junction strength between horizontal cells under local stimulus in intact retina (step1 → step2). For cutting experiments, retinas were cut before incubation (step2 → step1). **(D)** Coupling strength of illuminated horizontal cells in a ∼4 mm^2^ window (IN) and those in an adjacent unilluminated region (OUT). **(E)** Quantification of gap junction strength from 6 separate retinas treated as in **(D)**. *n* = 5–19 starter cells for each condition per retina. Error bars are SEM. **(F)** Coupling strength of illuminated horizontal cells in a ∼4 mm^2^ window (Cut_IN) and those in an adjacent unilluminated region (Cut_OUT) from cut retina. **(G)** Quantification of gap junction strength from 3 separate retinas treated as in **(F)**. *n* = 5–19 starter cells for each condition per retina. Error bars are SEM. Scale bars in **(A,D,E)**: 20 μm.

The strength of horizontal cell coupling is modulated by light in several species, with coupling stronger in dark-adapted than in light-adapted retinas (Teranishi et al., 1983; Piccolino et al., 1984; Godley and Wurtman, 1988; Dong and McReynolds, 1991; Xin and Bloomfield, 1999; He et al., 2000; Zhang et al., 2011; Dowling, 2012) Using the Pept2 method, we found that coupling strength is also light-dependent in mice: uptake of βALA into GFP-positive cells did not differ detectably between light- and dark-adapted retinas, but coupling strength was greater in the latter case (**Figures 6A,B**). Thus, electrical coupling of horizontal cells is modulated by illumination in mice as it is in other species.

The ability of the Pept2 method to assay coupling of large numbers of cells over a large area allowed us to ask whether the effect of light-adaptation was local or whether it spread to dark-adapted regions of the same retina. For this purpose, we mounted dark-adapted Sdk2-CreER; *pept2*^-/-^ retina infected with AAV-DiO-Pept2-GFP on a membrane filter with a small window in it, and placed it in a dish perfused with Ringer’s solution. We illuminated the entire retina with a dim background light (1 nW/cm^2^) and shone a bright spot (1 μW/cm^2^) through the window region (**Figure 6C**). The membrane filter was opaque and the periphery of the dish was lightproof, so only the portion of the retina within the window was illuminated. Nonetheless, coupling was equivalent for cells inside the window (light-adapted) and outside the window (dark-adapted; **Figure 6D**). Similar results were obtained in six separate experiments, with the coupling midway between that observed for fully light-adapted and fully dark-adapted retinas (**Figure 6E**). This result suggests that electrical synaptic strength is modulated based on the ‘averaged’ light level across a broad expanse of retina instead of its local level.

We considered two alternative explanations for how the result in **Figures 6D,E** might have arisen. A trivial explanation is that light scattered from the window or penetrated the opaque filter to illuminate the nominally dark-adapted retina. The more interesting possibility is that long-distance lateral connections mediate the effect. To distinguish these alternatives, we made cuts between the portions of the retina that lay inside and outside the window before illuminating the central region. In this case, coupling of cells inside the window was low whereas coupling of cells outside the window was equivalent to that in fully dark-adapted retina (**Figures 6F,G**). This result indicates that lateral connections lead to an equalization of coupling across the retina.

## Discussion

We developed a method for mapping electrical synaptic connections from genetically defined neurons based on a dipeptide transporter Pept2 and its fluorescent substrate βALA. Using transfection and virus infection, we tested the Pept2 method in cultured cells and the retina. In both case, we observed coupling patterns consistent with those reported previously and quantified gap junction strength. We then used the method to analyze light-dependent modulation of electrical connectivity.

### Choice of Pept2

We initially screened three channels, TRPV1, TRPA1, and P2X7, and two transporters, Pept1 and Pept2. In the cases of TRPV1, TRPA1, and P2X7, levels of fluorescent substrates in the transfected cells were low and electrically coupled cells were rarely detected. This observation could be explained in at least two ways: first, prolonged activation of these channels may lead to entry of large cations such as Ca^2+^, which can injure transfected cells (Jancsó et al., 1977; Meyers et al., 2003; Chen et al., 2009; Browne et al., 2013). We attempted to shorten the activation period or use a Ca^2+^ free incubation solution, but this had little effect. Alternatively, there is some evidence that the permeability of these channels to large cations requires activation of a secondary downstream pore (Bautista and Julius, 2008). In this case, introducing fluorophores into the transfected cells would require co-expression of the secondary channel or transporter, which may not have been present in the cells we tested.

In contrast, Pept1 and Pept2, which transport di- and tri-peptides and related drugs (Biegel et al., 2006; Newstead et al., 2011; Smith et al., 2013), were suitable for introduction of fluorophores into cells. Of the two, Pept2 proved to be superior, likely because of its higher affinity: Pept2 can bind and transport βALA into the cell efficiently even when the extracellular concentration is low. This low concentration of βALA causes low-level background, thus increases ratio of signal to noise (Daniel and Rubio-Aliaga, 2003; Zhang et al., 2004b; Biegel et al., 2006).

### Advantages of the Pept2 Method

Pept2 is an oligopeptide-proton symporter. It functions as a secondary active transporter by direct coupling to H^+^-ATPase, or by indirect coupling to an ATPase through other transporters, for instance, coupling to a Na^+^/H^+^ exchanger which in turn couples to a Na^+^/K^+^ ATPase (Beyenbach and Wieczorek, 2006; Sala-Rabanal et al., 2008; Newstead et al., 2011; Smith et al., 2013). Active transport allows Pept2 to transport βALA across the membrane against concentration gradient, allowing the intracellular concentration to reach a higher level than the extracellular concentration. Indeed, we observed brighter βALA signals in the transfected cells than in the bath solution. Once in the probe cell, βALA can diffuse readily through gap junctions as demonstrated by direct injection into HEK cells, cultured neurons and retinal neurons (**Figure 1** and data not shown). Results from microinjection indicate that ideal fluorescent tracer for gap junction coupling should be small and positively charged (Mills and Massey, 1998; Kanaporis et al., 2011). βALA has a net charge of +1, and a molecular weight of 432, comparable to 323 of Neurobiotin, a widely used tracer; and 579 of Po-pro-1, a recently reported tracer (Hoshi et al., 2006).

Compared with microinjection, the most widely used method for labeling electrically coupled neurons, the Pept2 method has at least two advantages. First, expressing Pept2 by transfection or virus infection enables us to label multiple cells at once, whereas cells must be targeted one by one for microinjection. Second, microinjection requires impaling a target cell with an electrode, whereas the Pept2 method is non-invasive, and maintains the integrity of the cell. Thus, the Pept2 method allows study of electrical coupling from small or fragile cells deeply buried in tissue, which poses challenges for microinjection.

To quantify the strength of coupling measured with the Pept2 method, we used diffusion equations, based on the finding that spread of dye approached steady state following 4 h of incubation. Previous methods for assessing coupling strength were based on measurements of fluorescence intensity at different times after dye injection (Mills and Massey, 1998; Abbaci et al., 2008). These methods do not require that steady state be reached, but do require multiple measurements, which are infeasible in many circumstances.

### Limitations of the Pept2 Method

Although the Pept2 method has advantages, it is currently limited in some ways. First, endogenous Pept2 is expressed by glial cells in the brain (Saito et al., 1996; Berger and Hediger, 1999; Dieck et al., 1999), and retina (**Supplementary Figure S1**). This endogenous Pept2 expression led to background signals that obscured signals in labeled neurons. We therefore performed studies in *pept2*^-/-^ mice so that probe cells and their electrically coupled cells could be visualized. This is obviously burdensome. Going forward, one could seek transporters that are not expressed in neuronal or glial cells, for instance, one derived from plants or fungi. Second, AMCA, the fluorophore used in this study, has low quantum yield compared with other fluorophores, such as fluorescein (Lavis and Raines, 2008). We used AMCA because βALA was commercially available. It should be possible, however, to synthesize the di-peptide conjugated to brighter fluorophores.

### Modulation of Electrical Coupling Between Horizontal Cells

The Pept2 method allowed us to study modulation of electrical coupling between neurons, a phenomenon that occurs in many neuronal circuits (Bargmann and Marder, 2013). Specifically, we analyzed light dependent modulation of electrical coupling between horizontal cells, which has been documented in several species (Teranishi et al., 1983; Piccolino et al., 1984; Godley and Wurtman, 1988; Dong and McReynolds, 1991; Xin and Bloomfield, 1999; He et al., 2000; Zhang et al., 2011; Dowling, 2012). A model for the role of modulation is as follows: In dim light, coupling is high, averaging signals from multiple photoreceptors and increasing the receptive field of the horizontal cells. This increase has the effect of enhancing the sensitivity of the retinal circuit at the expense of resolution. In bright light, coupling is decreased, sacrificing sensitivity for resolution (Masland, 2001; Bloomfield and Volgyi, 2009; Dowling, 2012).

We confirmed that light also modulates coupling of horizontal cells in mouse retina, and then asked whether the modulation was confined to the illuminated area. We found that electrical coupling between horizontal cells was modulated based on the ‘averaged’ light level across a large area of retina. This contrast-averaged adaptation is different from conventional contrast adaptation at the photoreceptor level, which is local: only directly illuminated photoreceptors became less sensitive to light. This spatial restriction generates the afterimage (dark sensation) when eyes are moved away from a bright spot (Virsu, 1978). Thus, both local and global contrast adaptation exist in the mouse retina.

We speculate that long-distance modulation of electrical coupling between horizontal cells results from long-range lateral connections within the retina. Light dependent modulation is known to depend on dopamine: light leads to activation of dopaminergic amacrine cells in the retina, and causes dopamine release. Dopamine binds to dopaminergic receptors in horizontal cells and weakens the electrical coupling (Lasater, 1987; DeVries and Schwartz, 1989). Dopaminergic amacrine cells extend long processes that can span up to half of the whole retina (Zhang et al., 2004a). Thus, we suggest that processes of dopaminergic amacrine cells underlie the ability of the retina to average light over a large area.

In summary, the Pept2 method provides a new tool for mapping electrical synaptic connectivity within neural circuits in the central nervous system. Combined with other techniques, such as functional imaging and gene profiling, it may also allow study of functional properties and molecular mechanism of electrical coupling.

## Materials and Methods

### Animals

JamB-CreER and Sdk2-CreER mice were generated in our lab and have been described previously (Kim et al., 2008; Krishnaswamy et al., 2015). JamB-CreER mice label J-RGCs, which are OFF-type direction selective RGCs responding to upward motion. Sdk2-CreER mice label horizontal cells, as well as specific subtypes of amacrine cells and RGCs. *pept2*^-/-^ mice, in which endogenous *pept2* expression was eliminated, were a kind gift from D. Smith (University of Michigan; Shen et al., 2003). Mice were maintained on a C57B6 background. All experiments were conducted in accordance with protocols approved by the Institutional Animal Care and Use Committees at Harvard University.

### Molecular Biology

Using standard molecular cloning techniques, coding sequences of Pept1 (mouse), Pept2 (human), and P2X7 (mouse) were inserted downstream of the CMV promoter in the plasmid of pCMV-N1-EGFP to generate pCMV-Pept1, pCMV-Pept2, and pCMV-P2X7. Constructs of pCMV-TRPV1 and pCMV-TRPA1 were gifts from R. Gaudet (Harvard University).

pCMV-Pept2-GFP was generated by fusing the coding sequence of Pept2 (without STOP codon) to the N terminal of EGFP in the plasmid of pCMV-N1-EGFP. pCMV-Pept2-p2a-GFP was generated by linking the coding sequence of Pept2 (without STOP codon) to EGFP using p2a sequence: 5′-CGGAAGCGGAGCTACTAACTTCAGCCTGCTGAAGCAGGCTGGAGACGTGGAGGAGAACCCTGGACCTA-3′ (Kim et al., 2011).

Adeno-associated virus plasmid pAAV-hSyn1-DiO-WPRE was a kind gift from B. Lowell (Harvard Medical School). The Pept2-GFP sequence was cloned into this plasmid. The final vector was verified by sequencing and packaged in serotype 2 capsids following procedures described previously (Guo et al., 2012).

### Cell Culture

HEK293 cells were seeded in 24-well plates and maintained in Dulbecco-modified Eagle’s minimal essential medium supplemented with 10% fetal calf serum. Plasmids were transfected into ∼80% confluent HEK cells using lipofectamine 2000 reagent (Life Technology). Five hundred nanogram of the channel or transporter DNA together with 500 ng pCMV-XFP DNA were transfected. For pCMV-Pept2-GFP and pCMV-Pept2-p2a-GFP, a total of 500 ng DNA was transfected.

After transfection, HEK cells were grown for another 1–2 days, and then dissociated by pipetting. Dissociated cells were mixed with untransfected HEK cells at a ratio of 1:100, replated in a 24-well plate, and grown for another day.

The assay for uptake and transfer was based on procedures described previously (Dieck et al., 1999). After aspiration of the culture medium, HEK cells were washed twice and preincubated for 10 min with HEPES-buffered saline (HBS): 145 mM NaCl, 5.4 mM KCl, 1.8 mM CaCl_2_, 1 mM MgCl_2_, 20 mM HEPES, and 20 mM glucose, pH = 7.2. The HBS was aspirated and the cells were incubated for 5 min–1 h for TRPV1, TRPA1, and P2X7 and 1–6 h for Pept1 and Pept2 with HBS containing fluorescent substrates. Substrate concentrations were: 100 μM for Yo-pro-1 and Po-pro-1 (Life Technology), 100 μM for Ethidium (Sigma) and 40 μM for βALA (Biotrend). Capsaicin (1 μM; Sigma), allyl isothiocyanate (300 μM; Sigma) or ATP (5 mM, Sigma) were included to activate TRPV1, TRPA1, and P2X7 channels, respectively. After incubation, the cells were rapidly washed twice with ice-cold HBS, and then fixed with 4% paraformaldehyde (PFA) at 4°C for 10 min. Fixed cells were imaged using a Nikon inverted fluorescence microscope.

To test the specificity of Pept2-dependent uptake of βALA, 8 mM Gly–Gln was applied together with 40 μM βALA for the incubation. To determine whether intercellular movement of βALA depended on gap junctions, HEK cells were preincubated with HBS containing 100 μM CBX or MFA for 10 min before βALA was applied.

### Quantification of Gap Junction Strength from *In Vitro* Assays

The diffusion of βALA from probe cells to coupled cells can be described by the following diffusion equations:

(1) $$J\left(x,\;t\right)=\;-D\frac{\partial }{\partial x}C(x,\;t)$$

(2) $$\frac{\partial }{\partial t}C\left(x,\;t\right)=\;-\frac{\partial }{\partial x}J\left(x,\;t\right)-k_{\text{out}}C\left(x,\;t\right)+k_{\text{in}}C_{\text{solution}}$$

We define the probe cell as having position *x* = 0, and number other cells by their distance from the probe cell. For example, cells directly coupled to the probe cell are designated *x* = 1, cells coupled to directly coupled cells are designated *x* = 2, cells coupled to *x* = 2 cells are designated *x* = 3 and so on (**Figure 4A**). *C*(*x, t*) indicates the concentration of βALA in a cell at position *x* at time *t*, and C_solution_ is the concentration of βALA in the incubation solution. βALA was left in the extracellular solution throughout the diffusion step.

Equation 1 gives, at time point *t*, the amount of substrate that diffuses between adjacent cells is proportional to the concentration difference between the two cells. *D* is the diffusion coefficient, *J*(*x, t*) is the flow of βALA at cell at position *x* at time *t* with units of concentration per unit time, which measures the amount of βALA diffused from cell at position *x* to adjacent cells (*x* + 1 or *x* – 1) in a unit time.

Equation 2 describes change of substrate concentration in a cell at position *x* at time *t*. This change has three sources, which are represented on the right side of the equation. The first is the flow of substrates from adjacent cells. Second, some βALA may be to the extracellular solution, for example through transporters such as multidrug resistant proteins. k_out_ indicates the rate constant of this export. Third although βALA is a specific substrate for Pept transporters, a very small amount may enter the cell non-specifically. k_in_ is the rate constant of this process.

Considering cells far away from the probe cells (x →∞), these cells receive little flow of βALA (J → 0). Thus, when the concentration of βALA is steady in these cells (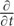 C = 0), based on Eq. (2), we have

(3) $$k_{\text{out}}C(x\to \infty )=k_{\text{in}}C_{\text{solution}}$$

We define this *C*(x →∞) as C_bg_.

At steady state when concentration of βALA in the cells doesn’t change with time, we have

(4) $$\frac{\partial }{\partial t}C\left(x,\;t\right)=0$$

Solving Eqs (1) and (2) at steady state, we get

(5) $$C\left(x\right)=\left(C_{0}-C_{bg}\right)e^{-\lambda x}+C_{bg}\;$$

Where, *C*_0_ is the concentration of βALA in the probe cell. Equation (5) has the form of an exponential decay, with decay constant

(6) $$\lambda =\sqrt{\frac{k_{out}}{D}}$$

We assume the measured fluorescent intensity (*I*) is proportional to the concentration (*C*) of βALA, and we get

(7) $$I\left(x\right)=\left(I_{0}-I_{bg}\right)e^{-\lambda x}+\;I_{bg}$$

Normalizing fluorescent intensity to that of probe cell I_0_, we get

(8) $$I_{normalized}\left(x\right)=\left(1-\frac{I_{bg}}{I_{0}}\right)e^{-\lambda x}+\frac{I_{bg}}{I_{0}}$$

We fit data with this curve to get the decay constant λ.

Gap junction strength *S* can then be defined as the proportion of substrate in the probe cell diffuses into the directly coupled cells:

(9) $$S=\;\frac{\left(C_{0}-C_{bg}\right)e^{-\lambda (x+1)}}{\left(C_{0}-C_{bg}\right)e^{-\lambda x}}=\;e^{-\lambda x}$$

### Retinal Assays

Using methods described previously, AAV-hSyn1-DiO-Pept2-GFP was delivered subretinally (Samuel et al., 2014) to Sdk2-CreER; *pept2^-/-^* animals to label horizontal cells, and delivered intravitreally (Hong et al., 2011) to JamB-CreER; *pept2^-/-^* animals to label J-RGCs. For virus injection, adult animals were anesthetized by intraperitoneal injection of ketamine/xylazine. A 30^1/2^G needle was used to make a small hole in the temporal eye, below the cornea, and 2 μL AAV-DiO-Pept2-GFP was injected with a Hamilton syringe and a 33G blunt-ended needle. Injected animals were euthanized and their retinas were dissected 4 weeks following injection.

Mice were dark adapted for at least 1 h prior to euthanasia. Retinas were dissected under infrared illumination in Mouse Ringers’ solution (140 mM NaCl, 2.5 mM KCl, 2 mM CaCl_2_, 1 mM MgCl_2_, 22 mM NaHCO_3_, and 10 mM glucose) oxygenated with 95% O_2_, 5% CO_2_ at room temperature. The retina was then moved to a 6 cm petri dish perfused with oxygenated Mouse Ringers’ at 32–34°C. βALA was added to the Mouse Ringers’ to a final concentration of 40 μM, and the incubation was continued for ∼4 h. After incubation, the retina was rinsed twice with ice-cold oxygenated Mouse Ringers’, and then fixed with 4% PFA at 4°C for 30 min. The retina was then processed for immunostaining and imaging.

To study light-dependent modulation of gap junction strength, a ∼2 mm aperture was cut into an opaque nitrocellulose filter (HABG01300, Millipore) and the dissected retina was mounted on the filter. The filter was then mounted into a lightproof 6 cm petri dish. Stimulation from a digital-light-processing projector (Dell) was focused onto the aperture. Stimulation intensity was 1 μW/cm^2^. Background light intensity was 1 nW/cm^2^. Thirty minutes after the onset of light stimulation, βALA was added to the Mouse Ringers’. After incubation, the retina with filter paper was rapidly washed twice with ice-cold oxygenated Mouse Ringers’ and fixed with 4% PFA at 4°C for 30 min. Following additional washes in PBS, the part of the retina within the aperture was separated from the rest by a sharp blade, and the two parts were processed for immunostaining and imaging.

To test whether the transfer of βALA was through gap junction, 100 μM MFA was applied 10 min before adding βALA.

### Immunohistochemistry

Fixed retinas were either frozen and sectioned at 20 μm in a cryostat or stained as whole mounts. Retina sections or whole mounts were incubated in PBS with 3% donkey serum and 0.3% Triton X-100 for blocking, followed by primary antibodies for ≥24 h at 4°C and secondary antibodies for ∼4 h. Retinas were then washed with PBS and mounted in Fluoromount G (Southern Biotech).

Primary antibodies used in this study were: rabbit anti-GFP (1:1000, Millipore); rabbit and mouse anti-calbindin (1:2000, Swant and 1:100, Swant); goat anti-choline acetyltransferase (ChAT), (1:400, Millipore); mouse anti-Brn3a (1:1000, Millipore); goat anti-Chx10 (1:200, Santa Cruz); mouse anti-AP2 (1:1000, DSHB); mouse anti-HCN4 (1: 1000, clone N114.10); rabbit anti-PKC alpha (1:1000, P4334 in Sigma); rabbit anti-secretagogin (1:4000, Biovendor); rabbit anti-arrestin (1:200, Milipore); rabbit anti-glutamine synthetase (1:1000, BD Biosciences); rabbit anti-sox9 (1:1000, Chemicon); mouse anti-connexin 43 (1:50, Life Sciences); rabbit antibody to Dab1 (a kind gift from B. Howell, SUNY Upstate Medical University). Secondary antibodies were conjugated to DyLight 649 (Jackson ImmunoResearch), Alexa Fluor 568, or Alexa Fluor 488 (Invitrogen) and used at 1:500.

### Imaging and Quantification of Signals

Retinal images were taken with Zeiss LSM-710 confocal microscope using 405, 488, 568, and 647 lasers with a step size of 0.5 μm and a 40X NA 1.3 lens. Images were then analyzed using ImageJ (NIH) software.

To quantify electrical synaptic strength between horizontal cells, the βALA signal in the starter GFP-positive horizontal cell was measured as *I*_0_. Horizontal cells surrounding this cell were identified by immunostaining for the marker calbindin. Because horizontal cells form a mosaic in mouse retina, we took the six horizontal cells that were closest to the starter cell as directly coupled cells, and measured βALA signals in them as *I*_1_*_._* The electrical synaptic strength *S* was defined as the proportion of substrate in the starter cell diffuses into the directly coupled cells. Adjusting for the background signals, we calculated the electrical synaptic strength as following:

(10) $$S=\frac{AveragedI_{1}-I_{bg}}{I_{0}-I_{bg}}$$

## Author Contributions

MQ and JRS conceived the project, designed experiments, and wrote the manuscript. MQ performed experiments and analyzed data.

## Conflict of Interest Statement

The authors declare that the research was conducted in the absence of any commercial or financial relationships that could be construed as a potential conflict of interest.

## Supplementary Material

Figure S1 **Muller glial cells express endogenous *pept2* and take βALA. (A–C)** βALA uptake in wild type retina **(A)**, wild type retina treated with Gly–Gln **(B)** and pept2^-/-^retina **(C)**. Sections were stained with antibody to glutamine synthetase and sox 9, which are molecular markers for Muller glia cells. Scale bar: 20 μm.

Figure S2 **Normal retinal architecture in *pept2*^-/-^ animals.** Sections of *pept2*^-/-^ retinas stained with antibodies to cell-type specific markers. No differences between wild type and *pept2*^-/-^ retinas were detected: Brn3a labels most RGCs; AP2 labels all amacrine cells; Chx10 labels all bipolar cells; Anti-ChAT labels somas and dendrites of starburst amacrine cells; Anti-protein kinase C (PKC) labels rod bipolar cells and a small subset of amacrine cells; Anti-disabled (DAB) labels AII amacrines; Anti-Secretagogin labels subsets of bipolar cells; Anti-calbindin labels horizontal cells, subsets of RGCs and amacrine cells, including starburst amacrine cells; Anti-HCN4 labels type 3a bipolar cells; Anti-Arrestin labels cone photoreceptors. Scale bar: 20 μm.
