# Supplementary figures and images for: Genetic Method for Labeling Electrically Coupled Cells: Application to Retina
Source: Front Mol Neurosci. 2016 Jan 7;8:81. doi: 10.3389/fnmol.2015.00081 (PMC4703850; doi:10.3389/fnmol.2015.00081)

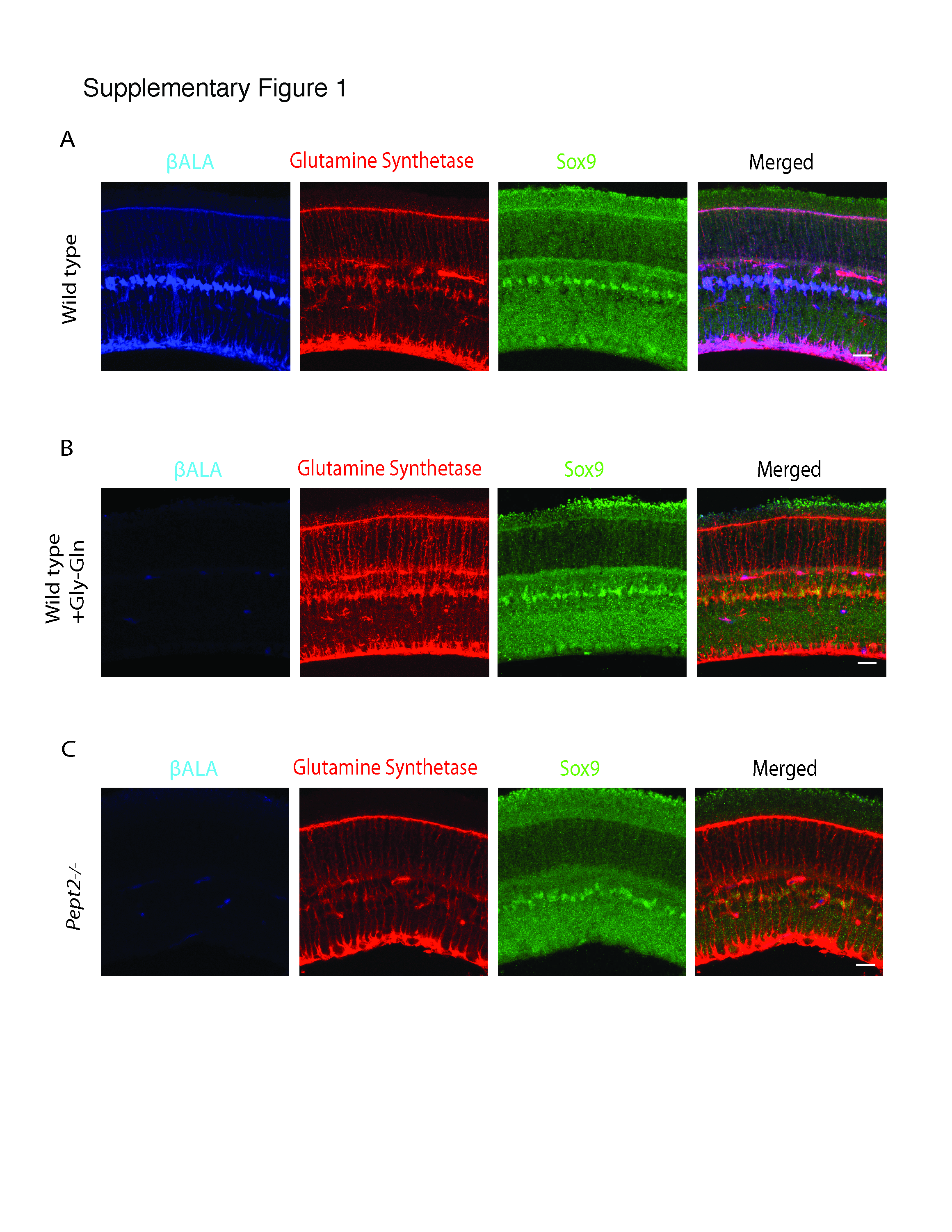

Supplement: Figure S1 — Muller glial cells express endogenous pept2 and take βALA. (A–C) βALA uptake in wild type retina (A), wild type retina treated with Gly–Gln (B) and pept2-/-retina (C). Sections were stained with antibody to glutamine synthetase and sox 9, which are molecular markers for Muller glia cells. Scale bar: 20 μm. [file Image_1.TIFF]

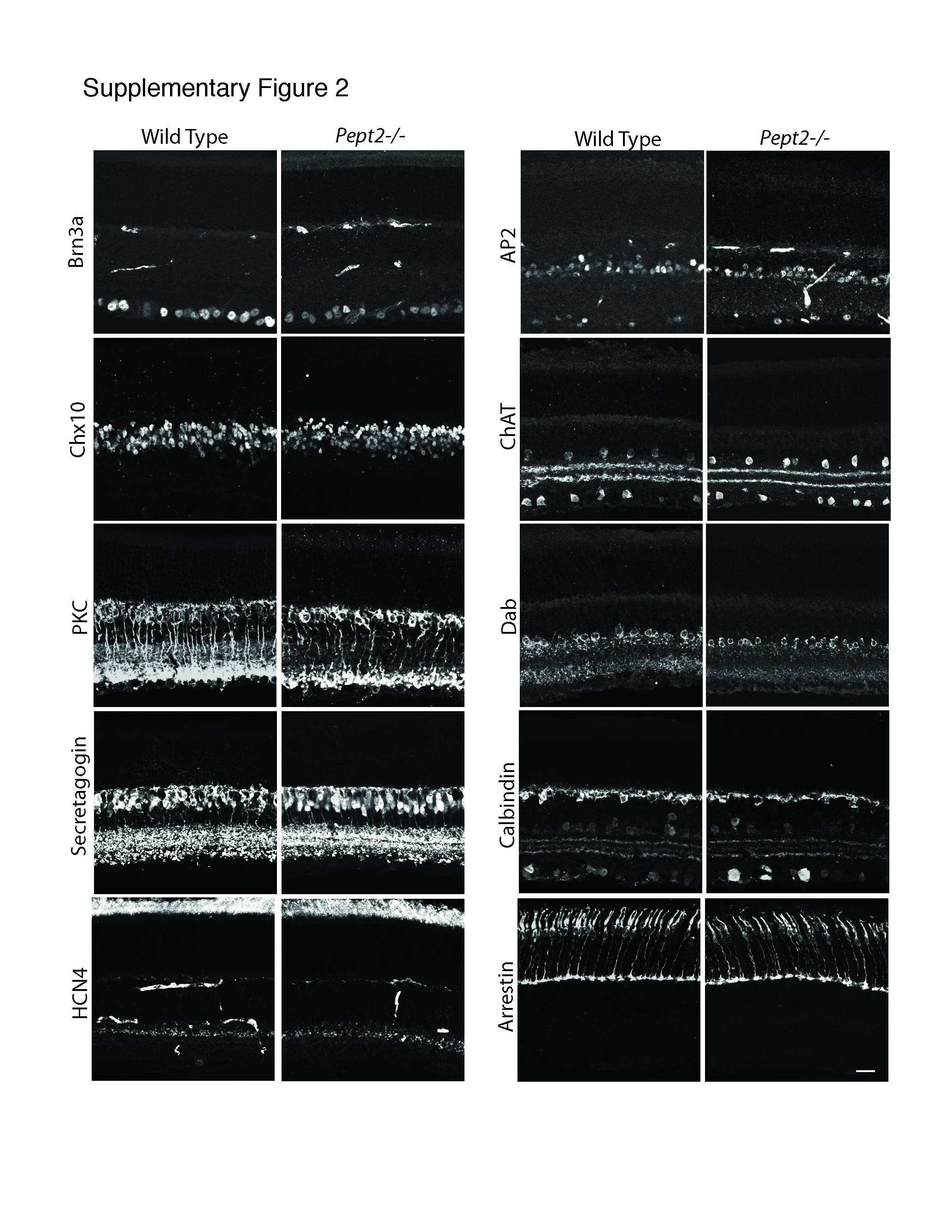

Supplement: Figure S2 — Normal retinal architecture in pept2-/- animals. Sections of pept2-/- retinas stained with antibodies to cell-type specific markers. No differences between wild type and pept2-/- retinas were detected: Brn3a labels most RGCs; AP2 labels all amacrine cells; Chx10 labels all bipolar cells; Anti-ChAT labels somas and dendrites of starburst amacrine cells; Anti-protein kinase C (PKC) labels rod bipolar cells and a small subset of amacrine cells; Anti-disabled (DAB) labels AII amacrines; Anti-Secretagogin labels subsets of bipolar cells; Anti-calbindin labels horizontal cells, subsets of RGCs and amacrine cells, including starburst amacrine cells; Anti-HCN4 labels type 3a bipolar cells; Anti-Arrestin labels cone photoreceptors. Scale bar: 20 μm. [file Image_2.TIFF]
